# Amphotericin B resistance in *Lomentospora prolificans* is associated with a soluble cell wall component

**DOI:** 10.1128/aac.00915-26

**Published:** 2026-09-16

**Authors:** Nina T. Grossman, Arturo Casadevall

**Affiliations:** 1 Department of Molecular Microbiology and Immunology, Johns Hopkins School of Public Health 25802 , Baltimore, Maryland, USA; 2 Divison of Infectious Diseases of the Johns Hopkins School of Medicine 1500 , Baltimore, Maryland, USA; University Children's Hospital Münster, Münster, Germany

**Keywords:** *Lomentospora prolificans*, amphotericin B, resistance

## Abstract

*Lomentospora prolificans* is a pathogenic filamentous fungus that causes disease primarily in people with severely compromised immune systems. It is pan-resistant to antifungal drugs, but the mechanism of its resistance to amphotericin B (AMB) is unknown. In this study, our goal was to investigate the mechanism of resistance to AMB of *L. prolificans*. The AMB susceptibility of *L. prolificans* protoplasts were measured using broth microdilution. *L. prolificans*, either intact, homogenized, or fractionated, was incubated with AMB in broth. The same activity was carried out with *Aspergillus fumigatus* as a control. These broths were then used to prepare microdilution plates with *Saccharomyces cerevisiae* to determine the activity of the conditioned AMB. AMB was 16-fold more effective at inhibiting the growth of *L. prolificans* protoplasts than conidia, but only 2-fold more effective against *A. fumigatus* protoplasts than conidia. Incubation of *L. prolificans* hyphae with AMB in media diminished drug activity to a much greater extent than *A. fumigatus*, with an 8-fold greater fungal mass of the latter required to achieve the effect of the former. Homogenization and fractionation of *L. prolificans* revealed that the factor inhibiting AMB activity was soluble with a mass >100 kDa. DNase, trypsin, proteinase K, amyloglucosidase, SDS, and 0.22 μm filtration had no effect on the AMB resistance factor, while treatment with urea and acetonitrile inactivated it. In summary, we report a different mechanism for AMB resistance for *L. prolificans* based on the existence of a substance residing in the cell wall that can eliminate the antifungal activity of AMB.

## INTRODUCTION

*Lomentospora prolificans* is a pathogenic filamentous fungus that causes disease primarily in people with severely compromised immune systems. While *L. prolificans*-related disease has a fairly low incidence, with one study estimating 0.53 cases per million people annually in Australia (1), such cases have remarkably high fatality rates. Globally, among patients with malignancy, which is a common risk factor for invasive lomentosporiosis, all-cause mortality can be as high as 85.7% (2). The primary reason for the deadly nature of *L. prolificans* infections is that the organism is pan-resistant to conventional antifungal drugs, making it very difficult to treat (3, 4).

*L. prolificans* represents one of the few fungal species that show inherent *in vitro* resistance to all three major classes of antifungal drugs—azoles, echinocandins, and polyenes. An analysis of mostly clinical *L. prolificans* isolates found that the minimum concentration of drug necessary to successfully inhibit the growth of all but the most resistant 10% of the isolates was greater than the highest concentration of drug tested for all eight of the antifungals tested (4). In this characteristic, it surpasses even isolates of the notoriously resistant *Candidozyma auris* (also known as *Candida auris*), 91%, 12%, and 7% of which are resistant to fluconazole, amphotericin B (AMB), and echinocandins, respectively (5). However, only one mechanism of resistance has thus far been identified in *L. prolificans*—an *FKS1* protein sequence that prevents efficient binding by echinocandin drugs (6, 7).

Among most human fungal pathogens, AMB resistance is rare and poorly understood. The mechanisms associated with reduced susceptibility to amphotericin B tend to fall into three broad categories. The first and most reported of these is alteration to the lipid composition of the cell membrane, generally from loss-of-function mutations in genes in the ergosterol synthesis pathway. This results in lower cell membrane ergosterol content, thereby providing less target for the drug to bind (8). The second category of AMB resistance mechanisms is the increased production of substances capable of binding amphotericin B and preventing it from reaching the cell membrane. Such substances include β-glucans, which are found in the extracellular matrix of biofilms of Saccharomycotina yeasts and appear to bind not just AMB but also fluconazole (9–12), and melanin, which is hypothesized to sequester AMB away from the cell membrane and also impede the drug’s access to its target by reducing cell wall pore size in *Cryptococcus neoformans* (13–16). Finally, the third set of hypothesized mechanisms of AMB resistance compensates for the oxidative damage done by the drug, either through overexpression of catalase or upregulation of heat shock proteins (17–19).

As incomplete as the understanding of mechanisms of amphotericin B activity and resistance is, the current knowledge on the mechanism of amphotericin B resistance in planktonic *L. prolificans* is even sparser. Two publications document successful attempts to interfere with the capacity of *L. prolificans* to melanize, the first by random mutagenesis and the second through the creation of knockouts of three genes in the melanin synthesis pathway (20, 21). Both groups found that the mutants remained resistant to amphotericin B treatment, even in the absence of melanin. Another study shows increases in SOD and catalase activity in response to AMB exposure in *L. prolificans*, as well as *Scedosporium minutisporum* and *Scedosporium aurantiacum*, the latter of which shows much lower rates of resistance than *L. prolificans*, suggesting that superoxide dismutase and catalase activity alone are probably not responsible for the remarkable resistance of *L. prolificans* (4, 22). Additionally, AMB synergizes with echinocandins against *L. prolificans* (23, 24), suggesting that resistance to the former may be related to the cell wall, synthesis of which is inhibited by echinocandins.

In this paper, we sought to understand the potential role of the cell wall in the resistance of *L. prolificans* to amphotericin B. After noting that removal of the cell wall rendered *L. prolificans* protoplasts susceptible to AMB, we established that a soluble component of the *L. prolificans* cell wall could remove or inactivate AMB in solution.

## RESULTS

To determine whether the cell wall contributes to antifungal resistance in *L. prolificans,* we examined the effect of cell wall removal on its susceptibility to AMB. Using a broth microdilution plate, we measured the minimum inhibitory concentration (MIC) of AMB against protoplasts and intact conidia of both *L. prolificans* and *A. fumigatus*. We found that AMB was 16-fold more effective against *L. prolificans* protoplasts than against its conidia (MIC 1 μg/mL vs 16 μg/mL, respectively, Table 1). In contrast, *A. fumigatus* showed only one dilution of difference between protoplasts and conidia (MIC 0.5 μg/mL vs 0.25 μg/mL). This suggested that the *L. prolificans* cell wall was essential to its unusual ability to grow in high concentrations of AMB and that the capacity of the cell wall to augment AMB resistance was not a common characteristic among filamentous fungi.

**TABLE 1:** AMB susceptibility of *L. prolificans* and *A. fumigatus* conidia and protoplasts by broth microdilution plate (modified CLSI methodology)

Species/condition	AMB MIC
*L. prolificans* 3.1 conidia	16 μg/mL
*L. prolificans* 3.1 protoplasts	1 μg/mL
*A. fumigatus* MYA-3626 conidia	0.25 μg/mL
*A. fumigatus* MYA-3626 protoplasts	0.5 μg/mL

We theorized that the *L. prolificans* cell wall could be acting as a drug sink—binding the AMB and preventing it from reaching the cell membrane. To test this hypothesis, *L. prolificans* hyphal mass was added to RPMI 1640 broth with AMB in various concentrations, shaken for an hour at 37°C, and then filtered out using a 0.22 μm filter. This sterile conditioned medium was then used to prepare a broth microdilution drug susceptibility plate with a *Saccharomyces cerevisiae* strain of known AMB susceptibility (Fig. 1). Accordingly, the MIC of the *S. cerevisiae* was used to determine how much drug activity remained in the media after incubation with *L. prolificans*, rather than the drug susceptibility of the yeast. *S. cerevisiae* was selected because it is susceptible to AMB and safe and easy to work with. Adding increasing concentrations of *L. prolificans* to the media diminished AMB activity in a dose-dependent manner, with 0.5, 1.0, and 1.5 mg/mL of fungal mass roughly multiplying the initial concentration of AMB necessary to inhibit yeast growth by, on average, two, four, and eight, respectively (Fig. 2). For comparison, the same process was conducted with *S. apiospermum* (MIC = 2 µg/mL) and *A. fumigatus*. While the addition of either of these fungi was sufficient to diminish the drug activity in the conditioned media, a much greater mass of both fungi was required to achieve the same effect as conditioning with *L. prolificans*. While 0.5 mg/mL of *L. prolificans* fungal mass reduced the drug activity by one dilution, 1.5 mg/mL of *S. apiospermum* and 4 mg/mL of *A. fumigatus* were necessary before the same reduction in drug activity was observed.

**Fig 1:**
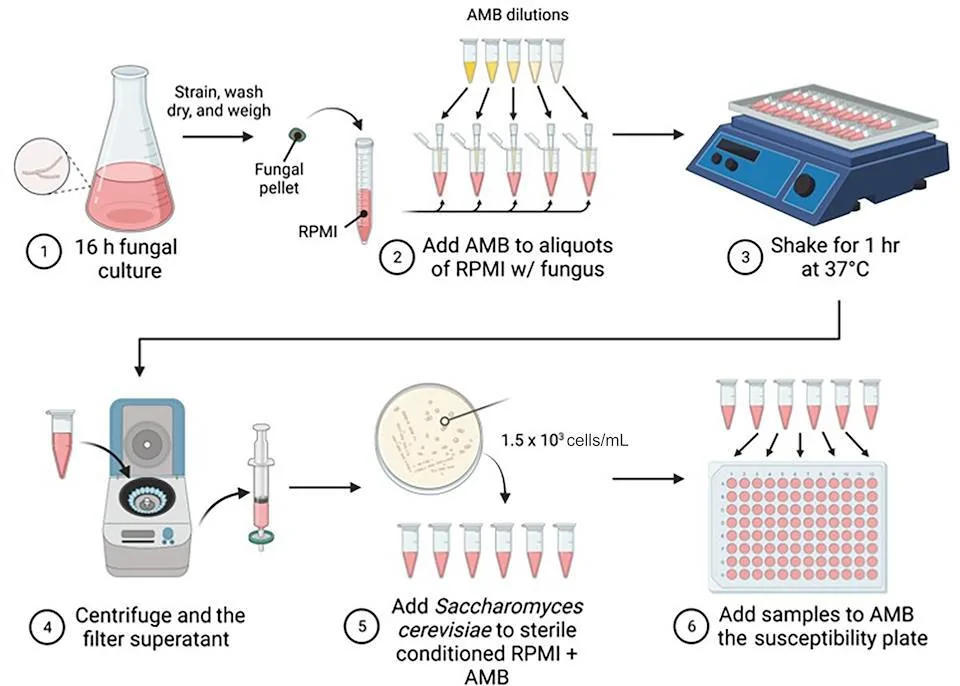
Steps involved in preparing the drug activity assay plate. Figure created using BioRender.com.

**Fig 2:**
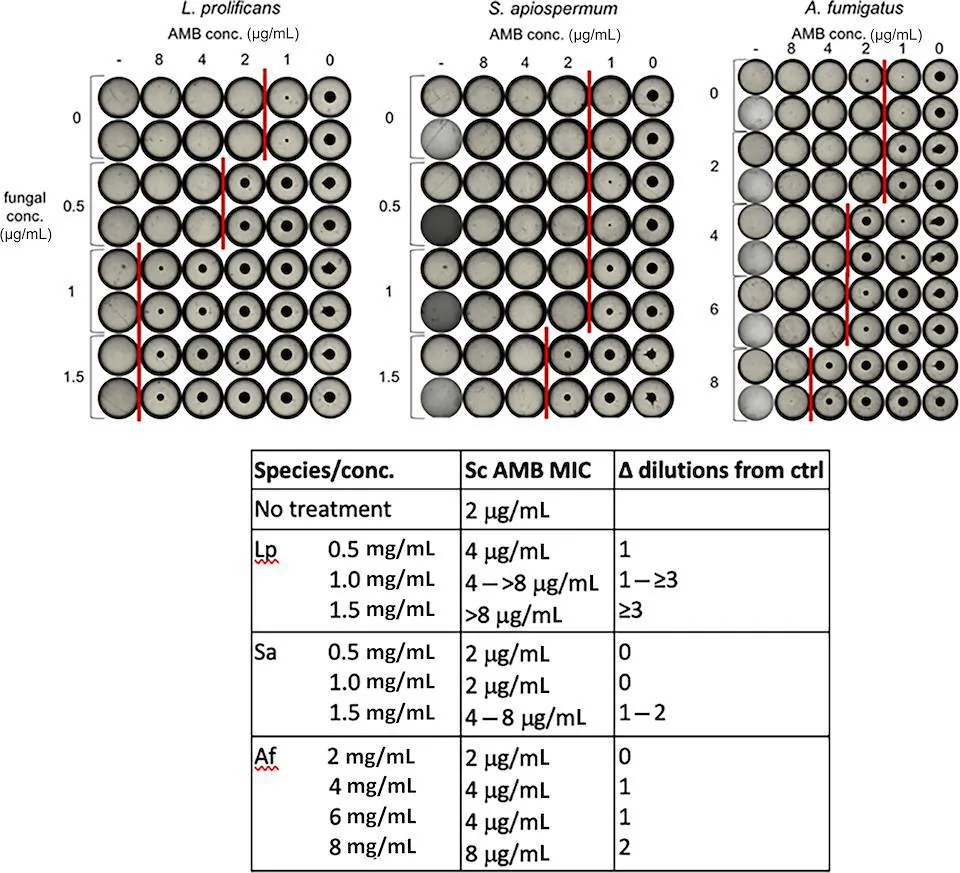
Reduction in AMB activity against *Saccharomyces cerevisiae* from incubation with intact *L. prolificans*, *S. apiospermum,* and *A. fumigatus* hyphae. Various concentrations by weight of the listed fungi were shaken with AMB in RPMI before the medium was sterilized and used in an AMB susceptibility plate inoculated with *S. cerevisiae*. Growth beyond that shown in fungal-free controls (denoted by “-”) indicates depletion of drug activity. Red lines separate wells with growth from wells without growth. Similar results were obtained in five biological replicates in technical duplicates for *L. prolificans* and three biological replicates in technical duplicates for *S. apiospermum* and *A. fumigatus*.

Having established that the presence of *L. prolificans* reduced AMB activity to an extent not observed with other filamentous fungal pathogens that are more susceptible to AMB, we next explored whether this effect was contingent on direct contact between the fungus and the media. Accordingly, the drug activity assay was repeated with the alteration that the AMB was not added until after the fungus had been filtered out, such that the medium was conditioned, but the drug was not. Under these conditions, no reduction of drug activity was observed, indicating that the drug was interacting with the fungus itself, rather than with a secreted factor (Table 2).

**TABLE 2:** Drug activity assay results of RPMI 1640 incubated with *L. prolificans* before and after addition of AMB

Condition	*S. cerevisiae* S288C AMB MIC	Δ dilutions from control
Unconditioned media with unconditioned AMB	2 μg/mL	
Conditioned media with unconditioned AMB	1 μg/mL	−1
Conditioned media with conditioned AMB	>8 µg/mL	≥3

Given that the initial discrepancy in AMB susceptibility between *L. prolificans* conidia and protoplasts suggested a role for the cell wall in drug resistance, we tested whether the dramatic difference between the abilities of *L. prolificans* and *A. fumigatus* to alter drug activity would be maintained as protoplasts. By wet pellet mass, 4 mg/mL of *L. prolificans* protoplasts in media diminished drug activity by one dilution, while 8 mg/mL of *A. fumigatus* was necessary to produce the same effect (Table 3). By comparison, intact *L. prolificans* was about eight times as potent as intact *A. fumigatus* at reducing drug activity. Interestingly, the 2-fold difference in the drug activity-reducing capacity of *L. prolificans* and *A. fumigatus* protoplasts mirrors the single dilution difference in AMB susceptibility between *L. prolificans* and *A. fumigatus* protoplasts. The reduction of the effect of *L. prolificans* on drug activity compared to that of *A. fumigatus* following the removal of the cell wall reinforces the suggestion that the source of *L. prolificans* AMB resistance resides in the cell wall.

**TABLE 3:** Drug activity assay results of AMB incubated with *L. prolificans* or *A. fumigatus* protoplasts

Species/conc.	*S. cerevisiae* S288C AMB MIC	Δ dilutions from control
No treatment	1 μg/mL	
*L. prolificans* 3.1 protoplasts		
2 mg/mL	1 μg/mL	0
4 mg/mL	2 μg/mL	1
8 mg/mL	4 μg/mL	2
*A. fumigatus* MYA-3626 protoplasts		
4 mg/mL	1 μg/mL	0
8 mg/mL	2 μg/mL	1

In *C. albicans* biofilms, β-glucans have been linked to resistance to both AMB and fluconazole (9, 10, 12). To determine whether the same phenomenon could be occurring in *L. prolificans*, we tested whether incubation with *L. prolificans* could diminish azole activity in the same manner it does AMB activity. Incubation with *L. prolificans* had no effect on azole activity (data not shown).

Having established that *L. prolificans* can absorb or otherwise inactivate AMB, and that the cell wall is essential to this capacity, we evaluated whether cells must be alive and intact to produce this effect or whether the mere presence of the cellular materials was sufficient. Accordingly, the drug activity assay was conducted in parallel using an equal mass of either homogenized or intact cells. The resulting drug microdilution plate showed the same amount of growth in the homogenate- and intact cell-incubated media, demonstrating that both diminished AMB activity to the same extent (Table 4). This demonstrated that the effect on drug activity was not contingent on live, or even intact, *L. prolificans*.

**TABLE 4:** Drug activity assay results of AMB incubated with intact or homogenized *L. prolificans* hyphae

Species/concentration	*S. cerevisiae* S288C AMB MIC	Δ dilutions from control
No treatment	2 μg/mL	
*L. prolificans* 3.1 intact cells (1 mg/mL)	>8 µg/mL	≥3
*L. prolificans* 3.1 homogenized cells (1 mg/mL)	>8 µg/mL	≥3

To pursue the suggestion that a cellular component, as opposed to a cellular process, was responsible for abrogating the AMB drug activity, the *L. prolificans* cell homogenate was centrifuged and separated into three fractions: supernatant, a light-colored top layer of pellet, and a darker bottom layer of pellet. Each of these was then used in place of hyphae in the drug activity assay, with the concentration normalized to the sample’s starting hyphal mass. The supernatant had the same drug-diminishing effect as the unfractionated homogenate, while the top layer of pellet showed no effect on drug activity, and the bottom layer of pellet showed only minor effects on drug activity (Table 5). For comparison, the same experiment was conducted with an equal concentration (5 mg fungal mass/mL) of *A. fumigatus*, and none of the fractions showed any effect on drug activity. This was somewhat surprising, given the previous findings demonstrating the involvement of the cell wall in diminishing AMB activity, as most components of the cell wall would be expected to be present in the pellet, rather than the supernatant. The finding suggests that a soluble component attached to or contained within the cell wall but released by homogenization is responsible for the observed reduction in AMB activity.

**TABLE 5:** Drug activity assay results of AMB incubated with *L. prolificans* or *A. fumigatus* homogenate fractions

Species/fraction (1 mg starting culture/mL)	*S. cerevisiae* S288C AMB MIC	Δ dilutions from ctrl
No treatment	1 μg/mL	
*L. prolificans* 3.1 homogenate supernatant	>8 µg/mL	≥4
*L. prolificans* 3.1 homogenate pellet – top layer	1 μg/mL	0
*L. prolificans* 3.1 homogenate pellet – bottom layer	2 μg/mL	1
*A. fumigatus* MYA-3626 homogenate supernatant	1 μg/mL	0
*A. fumigatus* MYA-3626 homogenate pellet – top layer	1 μg/mL	0
*A. fumigatus* MYA-3626 homogenate pellet – bottom layer	1 μg/mL	0

To further elucidate the attributes of this “resistance factor,” the homogenate supernatant was fractionated by size. Passing through a 0.22 μm filter prior to use in the drug activity assay had no effect on efficacy compared to the unfiltered supernatant. Following this, all future drug activities were performed such that the cellular component of interest was added to media, then filter-sterilized, and then aliquoted for the addition of drug. This eliminated the need for centrifugation and filtration of individual samples (Step 4 in Fig. 1).

Further fractionation was performed using Amicon filters ranging from 3 kDa to 100 kDa in size, and the resulting fractions were used in a drug activity assay. Of these, only the >100 kDa fraction maintained an inactivating effect on AMB (Table 6).

**TABLE 6:** Drug activity assay results of AMB incubated with *L. prolificans* homogenate supernatant fractions fractionated by size filtration

*L. prolificans* 3.1 homogenate supernatant fraction (1 mg starting culture/mL)	*S. cerevisiae* S288C AMB MIC	Δ dilutions from control
No treatment	2 μg/mL	
Unfractionated supernatant	>2 µg/mL	≥1
<3 kDa	2 μg/mL	0
3–10 kDa	2 μg/mL	0
10–30 kDa	2 μg/mL	0
30–100 kDa	2 μg/mL	0
>100 kDa	>2 µg/mL	≥1

To further test the hypothesis that the resistance factor was contained within the cell wall, as suggested by the previous protoplast experiments, we tested the effects on drug activity of the supernatant and pellet left over after the generation of protoplasts by treatment with cell wall-digesting enzymes. While the pellet, which was divided into three layers by color, each of which was tested separately, showed no effect of AMB activity, the supernatant impaired drug activity to the same extent as the whole-cell homogenate supernatant, normalized by starting culture weight (Table 7). Together, this bolsters the suggestion that the resistance factor resides within the cell wall and is soluble in the supernatant upon being released either physically (through homogenization) or chemically (through cell wall digestion).

**TABLE 7:** Drug activity assay results of AMB incubated with fractions of material remaining the generation of *L. prolificans* protoplasts

Treatment	*S. cerevisiae* S288C AMB MIC	Δ dilutions from control
No treatment	2 μg/mL	
Unfractionated supernatant	>2 µg/mL	≥1
Post-protoplasting supernatant	>2 µg/mL	≥1
Post-protoplasting pellet – top layer	1 μg/mL	−1
Post-protoplasting pellet – middle layer	1 μg/mL	−1
Post-protoplasting pellet – bottom layer	1 μg/mL	−1

A series of treatments were tested for their ability to eliminate the action of the resistance factor. Treatment with DNase (0.27 U/μL), trypsin (0.00375%), proteinase K (0.2 mg/mL), amyloglucosidase (0.2%), zymolyase (0.05 mg/mL), and SDS (0.1%) had no impact on the action of the resistance factor (Table 8). However, treatment with urea (4 M) or acetonitrile (60%) prior to incubation in media for the drug activity assay eliminated the resistance factor’s effect, as did heating to 65°C for 1 h. Additionally, attempts to separate the sample into lipid and non-lipid fractions using two different methods (chloroform:methanol extraction adapted from Bligh and Dyer and BUME extraction as described by Löfgren et al.) resulted in no resistance factor effect in any resulting fraction (25, 26).

**TABLE 8:** Inactivation treatments grouped by their effect on the capacity of *L. prolificans* supernatant to counteract AMB activity

No difference (MIC_treated supernatant_ = MICuntreated_untreated supernatant_)	Complete elimination (MIC_treated supernatant_ = MICno_no supernatant_)
DNase (0.27 U/ μL)	Urea (4 M)
Trypsin (0.00375%)	BUME organic phase
Proteinase K (0.2 mg/mL)	BUME aqueous phase
Amyloglucosidase (0.2%)	Acetonitrile (60%)
SDS (0.1%)	Methanol/chloroform organic phase
0.22 μm filtration	Methanol/chloroform aqueous phase
Zymolyase 20T (0.05 mg/mL)	Heat (65°C for 1 h)

## DISCUSSION

In this paper, we report on the existence of a cell wall substance in *L. prolificans* that can eliminate the activity of AMB. This represents the first mechanism of AMB resistance identified in *L. prolificans*. While the mechanism by which the resistance substance interferes with AMB is unclear, it likely binds to the drug and prevents its binding to ergosterol.

Despite considerable effort, we could not isolate the AMB-inhibiting substance to sufficient purity for structural analysis. Nevertheless, we gleaned insight about its composition by exposing it to various conditions and then testing its amphotericin inhibiting property. While not definitive, our findings are consistent with the substance being a glycolipid. The suggestion is supported by the fact that, while most means of inactivation by chemical or enzymatic treatment were unsuccessful, separation into aqueous and organic phases eliminated its activity entirely. Previous work has shown that β-glucans are capable of facilitating AMB resistance, apparently by binding the drug and preventing it from reaching the cell membrane (9–12). It appears less likely that β-glucans are involved in this case, given that treatment with multiple β-glucanase enzymes did not diminish the drug resistance factor’s effects on AMB, and indeed digesting *L. prolificans* with lysing enzymes from *Trichoderma harzianum* served to release the factor into solution in an equivalent manner to homogenization. However, it is possible that the nature of the interaction between AMB and β-glucan, a polysaccharide, is similar to the interaction between AMB and the polysaccharide component of the resistance factor.

The revelation that the presence of *L. prolificans* is sufficient to reduce AMB activity has implications for clinical treatment. While *L. prolificans* rarely causes invasive infections, its presence in the lung appears to be more common, particularly in cystic fibrosis patients (27, 28). It is plausible that, even in a colonizing state, that *L. prolificans* may actively protect other pathogenic fungi from clearance with AMB treatment.

It is noteworthy that *S. apiospermum* displayed an ability to inactivate AMB in media that was significantly less than that of *L. prolificans*, but considerably greater than that of *A. fumigatus*, as, indeed, its susceptibility to AMB is between that of the two other species. It is possible that *Scedosporium* species trace their unusual AMB tolerance to the generation of a similar substance, either to a lesser extent than *L. prolificans* or in a less effective form.

In summary, AMB resistance in *L. prolificans* appears to be mediated by a soluble cell wall resistance factor. The factor appears to be a macromolecule with a mass >100 kDa, but we do not know if it is a single molecule or a complex. We note that this mechanism of AMB resistance is different from others described, implying the existence of cell wall substances that protect against this drug. While we were unable to purify this substance to a purity sufficient for structural studies, we note that its large size and likely heterogeneity could pose a formidable analytical problem. Nevertheless, the work described here provides a roadmap for future analytical studies and sets a precedent for the existence of new mechanisms of AMB resistance.

## MATERIALS AND METHODS

### Strains

The strains used in this work were *L. prolificans* strain 3.1 from Christopher Thornton, *Scedosporium apiospermum* strain IHEM 14462 from BCCM, *Aspergillus fumigatus* strain MYA-3626, and *Saccharomyces cerevisiae* strain S288C from the Susan Michaelis lab. Conidia were obtained from the filamentous fungi by inoculating frozen conidia into Sabouraud dextrose broth, growing them for 5–21 days shaking at 30°C, and then plating 2–3 mL on potato dextrose agar. After growing for 6–8 days, these plates were covered in Dulbecco’s phosphate-buffered saline (DPBS) without magnesium or calcium (Corning, Corning, NY) and scraped. The resulting conidia in DPBS were strained through a 70 μm cell strainer (Corning), then pelleted at 4,000 rpm, and washed three times. In the case of *A. fumigatus*, 0.05% Tween 20 was added to DPBS, which was then passed through a 0.22 μm Steritop filter (Millipore Sigma, Burlington, MA).

Cultures were grown by inoculating conidia into RPMI 1640 to a concentration of 10^6^ conidia/mL. These were grown shaking at 90 RPM for 16 h at 37°C, then strained through two layers of Miracloth (Millipore Sigma), and washed with Milli-Q water. Fungal biomass was squeezed dry with Kimwipes (Kimberly-Clark, Irving, TX) and weighed.

### Protoplasts

Protoplasts were generated using published methodology with minor modifications (21). Briefly, conidia were inoculated into Sabouraud dextrose broth to a concentration of 10^6^ conidia/mL and shaken at 30°C for ~16 h. This culture was strained through two layers of sterile Miracloth, washed with sterile Milli-Q water, and then the fungal biomass was transferred to sterile-filtered OM buffer with lysing enzymes from *Trichoderma harzianum* (1.2 M MgSO_4_·7H_2_O, 10 mM NaPO_4_ [pH 5.8], 5% lysing enzymes by weight) and shaken at 120 rpm at 30°C for 16–20 h. Digested cells in OM buffer were transferred into Oak Ridge centrifuge tubes, overlaid with chilled ST buffer (0.6 M sorbitol, 10 mM Tris-HCl [pH 7.0]), and centrifuged at 5,000 × *g* at 4°C for 15 min in a swinging-bucket rotor. Protoplasts were recovered at the OM/ST interface, transferred to an Oak Ridge centrifuge tube with cold STC buffer (1.2 M sorbitol, 10 mM Tris-HCl [pH 7.5], 10 mM CaCl_2_), and pelleted at 3,000 × *g* for 10 min at 4°C. Protoplasts were then washed and pelleted twice more.

### Antifungal susceptibility testing

Antifungal susceptibility testing was carried out using CLSI broth microdilution methodology (29). Briefly, conidia or protoplasts were collected as described above and diluted into RPMI with 1.2 M sorbitol and inoculated 1:1 into broth microdilution plates to a final concentration of 2 × 10^5^ (protoplasts) or 5 × 10^3^ (conidia) cells/mL and 0.6 M sorbitol. Plates were grown at 37°C in 5% CO_2_ for 3 days and then imaged. Protoplasts were inoculated to a higher concentration than conidia to attain visible growth. To ensure that the growth in tests of protoplast susceptibility reflected only protoplasts, and not any remaining intact cells, protoplasts were also diluted into RPMI without sorbitol and then inoculated into broth microdilution plates. Antifungal susceptibility testing was performed in biological triplicate and technical duplicate.

### Drug activity assay

The extent to which drug activity was reduced by the presence of fungal biomass or fractions thereof was assessed as follows. A known concentration (the equivalent of 1 mg of initial fungal mass/mL unless otherwise stated) of the substance of interest was added to RPMI 1640, which was then divided into aliquots to which a 100× dilution of AMB in DMSO, or DMSO alone, was added to a final 1× concentration of AMB. Each experiment also included RPMI 1640 without any of the substance of interest added. These samples were then shaken at 37°C for 1 h. In experiments in which the substance of interest could pass through a 0.22 μm filter, RPMI with the substance was sterile-filtered prior to aliquoting and adding drug. In experiments in which the substance could not pass through a filter, such as when using intact fungal cells or fungal homogenate, samples were sterile-filtered individually after shaking. *S. cerevisiae* strain S288C was then added to each sample to a final concentration of 1.5 × 10^3^ cells/mL, and samples were transferred to an empty U-bottom 96-well plate, which was incubated at 30°C for 2–3 days.

### Cell homogenization

Fungal biomass that had been squeezed dry and weighed was resuspended in Milli-Q water and added to screw-top tubes with 0.5 mm glass beads. This was shaken in a FastPrep-24 bead beater (MP Biomedicals, Irvine, CA) five times at 6 m/s for one min, with 1.5 min on ice in between. Fractionation of the supernatant was conducted by microcentrifugation for 15 min at 4°C.

### Digestions, treatments and fractionation of homogenate supernatant

DNase digestion was conducted using PureLink DNase I (Invitrogen, Waltham, MA), reconstituted following the manufacturer’s instructions. Reaction consisted of 80 μL of supernatant, 10 μL of DNase buffer (as provided), and 10 μL of DNase I. This was incubated at room temperature for 15 min. Trypsin digestion was performed using 7.5 μL of 0.05% Trypsin-EDTA (Thermo Fisher Scientific, Waltham, MA) with 100 μL of supernatant. Reaction was shaken at 37°C for 30 min, then halted using a final concentration of 0.033% soy bean trypsin inhibitor (Millipore Sigma). SDS treatment was conducted using a final concentration of 0.1% SDS. SDS was then removed from the sample using a 100 kDa Amicon filter (Millipore Sigma), and the retained fraction was used in the drug activity assay. Urea treatment was conducted at a final concentration of 4 M urea. Amyloglucosidase digestion was conducted using a 0.2% by volume aqueous solution of amyloglucosidase from *Aspergillus niger* in 25 mM sodium acetate at 37°C for 30 min. Proteinase K digestions were conducted with 0.2 mg/mL recombinant, PCR-grade proteinase K (Roche, Basel, Switzerland) in 30 mM Tris-HCl (pH 8.3) while shaking at 37°C for 1 h. Where specified, digestions were carried out instead with 10 mg/mL proteinase K-agarose. Digestion with zymolyase 20T (Thermo Fisher Scientific) was performed at 0.05 mg/mL of enzyme in M/15 buffer for 1 h at 37°C.

### Lipid extraction

Two different lipid extraction methods were utilized. The first was adapted from the Bligh and Dyer method (25). Briefly, 750 μL of 1:2 chloroform:methanol was added to 200 μL of sample and vortexed for 10 min. Then, 250 μL of chloroform was added, and the sample was vortexed for 1 min. Finally, 250 μL of water was added, the sample was vortexed for 1 min, and phases were collected. The second approach was the BUME method developed by Löfgren et al. in 2012 (26). The procedure was performed as written, with the only alteration being that it was carried out by hand rather than by robot.

## References

[1] Heath CH, Slavin MA, Sorrell TC, Handke R, Harun A, Phillips M, Nguyen Q, Delhaes L, Ellis D, Meyer W, Chen SCA. 2009. Population-based surveillance for scedosporiosis in Australia: epidemiology, disease manifestations and emergence of *Scedosporium aurantiacum* infection. Clin Microbiol Infect 15:689–693. doi:10.1111/j.1469-0691.2009.02802.x 19549223

[2] Seidel D, Meißner A, Lackner M, Piepenbrock E, Salmanton-García J, Stecher M, Mellinghoff S, Hamprecht A, Durán Graeff L, Köhler P, et al.. 2019. Prognostic factors in 264 adults with invasive *Scedosporium* spp. and *Lomentospora prolificans* infection reported in the literature and FungiScope. Crit Rev Microbiol 45:1–21. doi:10.1080/1040841X.2018.1514366 30628529

[3] Rodriguez-Tudela JL, Berenguer J, Guarro J, Kantarcioglu AS, Horre R, de Hoog GS, Cuenca-Estrella M. 2009. Epidemiology and outcome of *Scedosporium prolificans* infection, a review of 162 cases. Med Mycol 47:359–370. doi:10.1080/13693780802524506 19031336

[4] Lackner M, de Hoog GS, Verweij PE, Najafzadeh MJ, Curfs-Breuker I, Klaassen CH, Meis JF. 2012. Species-specific antifungal susceptibility patterns of *Scedosporium* and *Pseudallescheria* species. Antimicrob Agents Chemother 56:2635–2642. doi:10.1128/AAC.05910-11 PMC3346635 22290955

[5] Parnell LA, Dos Santos AR, Forsberg K, Lyman M, Misas E, Gade L, Sexton DJ, Litvintseva AP, Chow NA. 2026. Updated genomic epidemiologic description of *Candida* (*Candidozyma*) *auris*, United States. Emerg Infect Dis 32:728–739. doi:10.3201/eid3205.250760 PMC13174969 42044884

[6] Johnson ME, Katiyar SK, Edlind TD. 2011. New Fks hot spot for acquired echinocandin resistance in *Saccharomyces cerevisiae* and its contribution to intrinsic resistance of *Scedosporium* species. Antimicrob Agents Chemother 55:3774–3781. doi:10.1128/AAC.01811-10 PMC3147641 21576441

[7] Katiyar SK, Edlind TD. 2009. Role for Fks1 in the intrinsic echinocandin resistance of *Fusarium solani* as evidenced by hybrid expression in *Saccharomyces cerevisiae*. Antimicrob Agents Chemother 53:1772–1778. doi:10.1128/AAC.00020-09 PMC2681557 19258277

[8] Carolus H, Pierson S, Lagrou K, Van Dijck P. 2020. Amphotericin B and other polyenes—discovery, clinical use, mode of action and drug resistance. J Fungi 6:321. doi:10.3390/jof6040321 PMC7724567 33261213

[9] Nett JE, Sanchez H, Cain MT, Ross KM, Andes DR. 2011. Interface of *Candida albicans* biofilm matrix-associated drug resistance and cell wall integrity regulation. Eukaryot Cell 10:1660–1669. doi:10.1128/EC.05126-11 PMC3232725 21666076

[10] Nett J, Lincoln L, Marchillo K, Massey R, Holoyda K, Hoff B, VanHandel M, Andes D. 2007. Putative role of β-1,3 glucans in *Candida albicans* biofilm resistance. Antimicrob Agents Chemother 51:510–520. doi:10.1128/AAC.01056-06 PMC1797745 17130296

[11] Vediyappan G, Rossignol T, d’Enfert C. 2010. Interaction of *Candida albicans* biofilms with antifungals: transcriptional response and binding of antifungals to beta-glucans. Antimicrob Agents Chemother 54:2096–2111. doi:10.1128/AAC.01638-09 PMC2863626 20194705

[12] Mitchell KF, Taff HT, Cuevas MA, Reinicke EL, Sanchez H, Andes DR. 2013. Role of matrix β-1,3 glucan in antifungal resistance of non-albicans *Candida* biofilms. Antimicrob Agents Chemother 57:1918–1920. doi:10.1128/AAC.02378-12 PMC3623361 23318790

[13] van Duin D, Casadevall A, Nosanchuk JD. 2002. Melanization of *Cryptococcus neoformans* and *Histoplasma capsulatum* reduces their susceptibilities to amphotericin B and caspofungin. Antimicrob Agents Chemother 46:3394–3400. doi:10.1128/AAC.46.11.3394-3400.2002 PMC128748 12384341

[14] Jacobson ES, Ikeda R. 2005. Effect of melanization upon porosity of the cryptococcal cell wall. Med Mycol 43:327–333. doi:10.1080/13693780412331271081 16110778

[15] Eisenman HC, Nosanchuk JD, Webber JBW, Emerson RJ, Camesano TA, Casadevall A. 2005. Microstructure of cell wall-associated melanin in the human pathogenic fungus *Cryptococcus neoformans*. Biochemistry 44:3683–3693. doi:10.1021/bi047731m 15751945

[16] Ikeda R, Sugita T, Jacobson ES, Shinoda T. 2003. Effects of melanin upon susceptibility of *Cryptococcus* to antifungals. Microbiol Immunol 47:271–277. doi:10.1111/j.1348-0421.2003.tb03395.x 12801064

[17] Blum G, Hörtnagl C, Jukic E, Erbeznik T, Pümpel T, Dietrich H, Nagl M, Speth C, Rambach G, Lass-Flörl C. 2013. New insight into amphotericin B resistance in *Aspergillus terreus*. Antimicrob Agents Chemother 57:1583–1588. doi:10.1128/AAC.01283-12 PMC3623369 23318794

[18] Blum G, Perkhofer S, Haas H, Schrettl M, Würzner R, Dierich MP, Lass-Flörl C. 2008. Potential basis for amphotericin B resistance in *Aspergillus terreus*. Antimicrob Agents Chemother 52:1553–1555. doi:10.1128/AAC.01280-07 PMC2292529 18268082

[19] Blatzer M, Blum G, Jukic E, Posch W, Gruber P, Nagl M, Binder U, Maurer E, Sarg B, Lindner H, Lass-Flörl C, Wilflingseder D. 2015. Blocking Hsp70 enhances the efficiency of amphotericin B treatment against resistant *Aspergillus terreus* strains. Antimicrob Agents Chemother 59:3778–3788. doi:10.1128/AAC.05164-14 PMC4468683 25870060

[20] Ruiz-Díez B, Martínez-Suárez JV. 2003. Isolation, characterization, and antifungal susceptibility of melanin-deficient mutants of *Scedosporium prolificans*. Curr Microbiol 46:228–232. doi:10.1007/s00284-002-3858-7 12567248

[21] Al-Laaeiby A, Kershaw MJ, Penn TJ, Thornton CR. 2016. Targeted disruption of melanin biosynthesis genes in the human pathogenic fungus *Lomentospora prolificans* and its consequences for pathogen survival. Int J Mol Sci 17:444. doi:10.3390/ijms17040444 PMC4848900 27023523

[22] Mello TP, Oliveira SSC, Branquinha MH, Santos ALS. 2022. Decoding the antifungal resistance mechanisms in biofilms of emerging, ubiquitous and multidrug-resistant species belonging to the *Scedosporium/Lomentospora* genera. Med Mycol 60:myac036. doi:10.1093/mmy/myac036 35641191

[23] Yustes C, Guarro J. 2005. *In vitro* synergistic interaction between amphotericin B and micafungin against *Scedosporium* spp. Antimicrob Agents Chemother 49:3498–3500. doi:10.1128/AAC.49.8.3498-3500.2005 PMC1196235 16048969

[24] Martin-Vicente A, Guarro J, Capilla J. 2017. Does a triple combination have better activity than double combinations against multiresistant fungi? experimental *in vitro* evaluation. Int J Antimicrob Agents 49:422–426. doi:10.1016/j.ijantimicag.2016.12.015 28257903

[25] Bligh EG, Dyer WJ. 1959. A rapid method of total lipid extraction and purification. Can J Biochem Physiol 37:911–917. doi:10.1139/o59-099 13671378

[26] Löfgren L, Ståhlman M, Forsberg GB, Saarinen S, Nilsson R, Hansson GI. 2012. The BUME method: a novel automated chloroform-free 96-well total lipid extraction method for blood plasma. J Lipid Res 53:1690–1700. doi:10.1194/jlr.D023036 PMC3540853 22645248

[27] Jacobs SE, Wengenack NL, Walsh TJ. 2020. Non-*Aspergillus* hyaline molds: emerging causes of sino-pulmonary fungal infections and other invasive mycoses. Semin Respir Crit Care Med 41:115–130. doi:10.1055/s-0039-3401989 32000288

[28] Blyth CC, Middleton PG, Harun A, Sorrell TC, Meyer W, Chen SCA. 2010. Clinical associations and prevalence of *Scedosporium* spp. in Australian cystic fibrosis patients: identification of novel risk factors? Med Mycol 48 Suppl 1:S37–S44. doi:10.3109/13693786.2010.500627 21067328

[29] CLSI. 2008. Reference method for broth dilution antifungal susceptibility testing of filamentous fungi; approved standard-second. In A standard for global application developed through the Clinical and Laboratory Standards Institute consensus process. Clinical and Laboratory Standards Institute.

